# Outcomes and Benefits of Using Twice Daily Nebulised Hypertonic Saline in T2-Low and T2-High Asthma With Bronchial Hypersecretion: A Retrospective Cohort Study

**DOI:** 10.7759/cureus.118883

**Published:** 2026-10-31

**Authors:** Joseph Waddon, Rebekah Emmas, Erin McInally, Charlotte Hawkins, Katie Kemp

**Affiliations:** 1 General Surgery, Wrexham Maelor Hospital, Wrexham, GBR; 2 Cardiology, Wrexham Maelor Hospital, Wrexham, GBR; 3 Otolaryngology, Wrexham Maelor Hospital, Wrexham, GBR; 4 Respiratory Medicine, Wrexham Maelor Hospital, Wrexham, GBR

**Keywords:** asthma, bronchial hypersecretion, nebulised hypertonic saline, type-2 high asthma, type-2 low asthma

## Abstract

Objective

Patients with T2-low and T2-high asthma have limited treatment options. Our study aims to evaluate the efficacy of nebulised hypertonic saline (HS) as an adjunctive treatment in this cohort. Specifically, the study aims to evaluate whether HS is tolerated in these patients, given the historical concerns with HS-induced bronchoconstriction. Additionally, it aims to determine whether nebulised HS reduces the frequency of exacerbations and hospital admissions in patients with T2-low and T2-high asthma.

Methods

This retrospective cohort study reviewed adults with T2-low and T2-high asthma with a bronchial hypersecretion phenotype who underwent a successful trial of nebulised HS across two district general hospitals within Betsi Cadwaladr University Health Board in the United Kingdom between February 2023 and August 2025. We included patients who successfully tolerated the initial HS trial and remained on treatment for at least 12 months. Of the 48 people who passed the initial HS tolerance test, only 44 went on to continue the treatment for over a year. The clinical data, including asthma exacerbations requiring primary care treatment or hospital admissions, were compared between 12 months prior to starting treatment and 12 months after HS initiation. Pre- and post-treatment exacerbation frequencies and hospital admissions were compared using the paired Wilcoxon signed-rank test, with a statistical significance of p < 0.05.

Results

Of the 48 patients who passed the trial of nebulised HS, 44 remained on treatment after one year. The mean number of asthma exacerbations decreased significantly from 2.18 in the year before HS initiation to 1.20 in the year after treatment (p < 0.001). Mean hospital admissions due to acute asthma exacerbations significantly decreased, from 0.43 to 0.15 (p-value 0.006). Eleven patients (25%) were commenced on biologic therapy during the year following HS initiation.

Conclusion

Patients with T2-low and T2-high asthma who were started on long-term nebulised HS tolerated treatment well, and most remained on therapy at one year. Comparing the 12 months before and after starting HS, we found a statistically significant reduction in both exacerbations and hospital admissions. These findings suggest a potential role for nebulised HS as an adjunctive treatment in this patient group. However, this was an observational study, so we cannot establish causality, and confounding factors such as initiation of biologic therapy may have an influence. The findings are also limited as patients included are only from two hospitals, which results in reduced confidence in the generalisability of the findings to the wider population with the same diagnosis. Large-scale, prospective, controlled studies are required to confirm the efficacy of nebulised HS in this population.

## Introduction

Due to the variability in presentation and response to treatment, asthma, a chronic inflammatory disease of the airway, is described as a heterogeneous condition. In recent years, emergency hospital admissions due to asthma have been increasing, with over 40,000 admissions attributed to the disease in the UK in 2024 [1].

The pathophysiology of asthma can be broadly split into two categories: type 2 low (T2-low asthma) and type 2 high asthma (T2-high asthma) [2]. T2-low asthma is primarily defined as asthma with the absence of type 2 eosinophilic airway inflammation [2]. T2-high asthma, the most common subtype, is driven by the IL-4-, IL-5-, and IL-13-mediated pathway, which induces eosinophilic airway inflammation and results in mucus hypersecretion and airway obstruction. Type 2 inflammation is characterised using biomarkers, including FeNO and blood eosinophil count (thresholds typically between 250 and 300 cells/μL), alongside clinical and allergic features. There is no single biomarker threshold that independently establishes a diagnosis of T2-high asthma. T2-high asthma is associated with greater airway hyperresponsiveness, increased exacerbation rates, and more frequent admissions [3]. The management of this subtype is stepwise as per the British Thoracic Society (BTS) asthma management, and as treatment options become exhausted, there are currently several licensed biological therapy options [4].

The diagnosis and prevalence of T2-low asthma is less clear-cut, due to the absence of specific biomarkers and varying cutoffs for existing biomarkers. Often, patients diagnosed with T2-low asthma will have low blood eosinophil count (<150-300/µL), low FeNO (<20-25 ppb), and the absence of atopic symptoms or features. The prevalence of T2-low asthma in the UK is approximately 9.4%, and the inflammatory drivers are neutrophilic or paucigranulocytic [2,5]. Common T2-low phenotypes include late-onset, obesity-related, smoking, and non-atopic disease [2].

Tezepelumab is an approved biologic option for severe asthma and may provide benefit across a broader range of inflammatory phenotypes, including patients without marked eosinophilic inflammation [6]. Therefore, management of T2-low asthma is predominantly non-targeted. Current management includes optimisation of inhaled corticosteroids (ICS), consideration of long-term azithromycin, and addressing comorbidities such as obesity [2,7]. However, individuals with T2-low asthma are more likely to have a poor response to treatments such as ICS [2,8]. For the hypersecretory subgroup, mucoactive strategies can also be used [9]. Research has shown that patients diagnosed with this subtype are more likely to have poorer outcomes, including self-reported reduced symptom control and worsened quality of life [2].

Hypertonic saline (HS) is a sterile water solution with salt content higher than normal body fluid; inhalation of this provides an osmotic gradient which draws water away from the airway tissue and into the airway lumen [9]. The intended benefit of this is to hydrate the thick and sticky mucus, making it easier for the patient to loosen and clear their secretions. The majority of trial data surrounding the efficacy of this is within the context of bronchiectasis and cystic fibrosis; however, in the hypersecretory phenotype of asthma patients, the mucoactive mechanism is the same, as these patients also demonstrate a distinct mucus-hypersecretory phenotype [10,11]. Given the limited treatment options for T2-low and T2-high asthma and the mechanism overlap with bronchiectasis-type mucus retention, a mucoactive agent may provide significant benefit.

HS is an indirect bronchoconstrictor; while it does not act directly on airway smooth muscle, it can trigger narrowing through osmotic and inflammation-mediated pathways. Hence, there have been concerns over both the efficacy of the use of HS in the management of T2-low and T2-high asthma. The BTS guidelines (in the context of bronchiectasis) state that, when HS is first administered, FEV1 or peak expiratory flow rate should be measured both before and after administration. It also states that those with a higher risk of bronchial hyperreactivity, such as asthmatics, should be considered for pre-treatment with bronchodilators prior to the administration of HS [12]. However, if there is no significant fall in FEV1 post-administration of HS and it is tolerated by the patient, then regular or home use of HS can be used [10]. There is no nationally mandated numeric FEV1 fall threshold for the discontinuation of HS; current thresholds are derived from trial data and research protocols (Grade D evidence) [12]. The physiological rationale for concern is well-established in terms of its mechanism, but the specific protocol for a BD-dosed regimen in severe asthma with a hypersecretion phenotype is largely unstandardised.

Current research provides limited evidence on the use of 7% HS in patients with T2-low and T2-high asthma, and no dedicated trials have evaluated the regular use of HS in this patient population. Our study aims to address this evidence gap by evaluating the effects of twice-daily 7% HS on the frequency of asthma exacerbations and hospital admissions. We also aim to assess the tolerability of regular nebulised HS in this patient cohort.

## Materials and methods

Study design and setting

A retrospective cohort study was conducted to evaluate patients diagnosed with T2-low or T2-high asthma across two secondary care centres, Wrexham Maelor Hospital and Glan Clwyd Hospital in the United Kingdom, who were trialled on nebulised HS. Patients who were trialled on this between February 2023 and August 2025 were included in the study.

Ethical considerations

Formal research ethics committee approval was not required due to the study being a retrospective analysis and evaluation. Anonymised and routinely collected data were used, and the study was non-interventional. Individual patient consent was waived for the same reasons.

Patient selection

Patients were identified through the specialist respiratory department records of patients who were commenced on nebulised hypertonic salbutamol. Unique patient identifier numbers were used to ensure no duplicate records across the data sources were used. The health board did not consistently have a clear distinction between T2-low and T2-high patients; hence, the patients were analysed as one group.

Inclusion and exclusion criteria

Inclusion criteria included the following: patients ≥18 years old, diagnosis of T2-low asthma or T2- high asthma with a bronchial hypersecretion phenotype, and patients who had at least one year of nebulised HS. Exclusion criteria involved the following: patients on nebulised HS with an alternative diagnosis to T2-low/T2-high asthma, patients who had not had at least one year of treatment with HS, patients who did not pass the nebulised HS trial, and patients with incomplete records for essential variables required.

Data sources

Data were extracted from two electronic clinical systems used across these secondary care centres: Welsh Clinical Portal (WCP), which included access to patients' general practice records, and EPRO for specialist services clinic letters and documentation. These systems were used to extract the following information: baseline clinical data (age, gender, and diagnosis); details of nebulised HS (date of trial, outcome of trial, and whether treatment continued); exacerbation details (number of exacerbations experienced by individual patients in the year before the trial compared with the year after); and hospital admissions (number of hospital admissions experienced by individual patients in the year before the trial compared with the year after). Exacerbations were identified through hospital discharge letters/attendances and general practitioner (GP) records. An exacerbation constituted acute worsening of asthma symptoms requiring additional therapies. For the hospital admissions, the recorded diagnosis and management (nebulisers, steroids or antibiotics) were identified, and for the GP exacerbations, we were reliant on the correct coding by the GP for an asthma exacerbation.

Variables and definitions

The bronchial hypersecretion phenotype was defined as chronic bronchitis (productive cough on most days for ≥3 months in each of two consecutive years), and may have bronchial wall thickening but no diagnosis of bronchiectasis on their CT scan. Passing of the nebulised HS trial was defined as < 15% reduction in forced expiratory volume in one second (FEV1) immediately after and 20 minutes after administration of HS and return to within 5% of baseline FEV1 by 20 minutes post HS administration. This was evidenced through clinic letters detailing that patients had passed this trial.

Data management

The data were recorded in a structured spreadsheet using a predefined standardised data key to ensure consistency and accuracy in variable definitions and coding. Data cleaning was undertaken prior to formal analysis, which involved verification of discrepancies against source records and standardisation of variables. Any missing data were reassessed and handled according to our inclusion/exclusion criteria previously mentioned. Patients with missing data in key variables, such as unavailable GP records or incomplete records, were excluded from the study/analysis.

Statistical analysis

Statistical analysis of the data was performed using Jamovi (version 2.7.38; The Jamovi Project, Sydney, Australia). As the data were not normally distributed, the paired Wilcoxon signed-rank test was used to compare both pre- and post- treatment exacerbation frequencies and hospital admissions due to an exacerbation. Descriptive statistics (mean, median, standard deviation) were calculated for both time points. Statistical significance was set at p < 0.05.

## Results

A total of 49 patients were identified as having a nebulised HS trial with a diagnosis of asthma between February 2023 and August 2025. This included 31 (63%) women and 18 (37%) men, and 48 tolerated the trial and were initiated on regular HS treatment. From these 48 patients, a total of 44 patients continued the treatment for at least one year. The age range of patients included was 29-89 years, with the average being 64.

The average number of recorded exacerbations the year before treatment was 2.18, dropping to 1.20 (p < 0.001) in the year after starting nebulised HS treatment (Table 1). Figure 1 demonstrates the reduction in mean number of recorded asthma exacerbations one year prior to and one year after starting treatment. The maximum number of exacerbations dropped from 7 to 5. The combined number of exacerbations reduced from 96 to 53, a 45% reduction. The mean change in exacerbation frequency was -0.977 (95% CI: -1.45, -0.507). 

**Table 1:** Exacerbations of asthma recorded one-year before and after initiation of regular nebulised hypertonic saline

Number of asthma exacerbations	N	Mean	Median	SD	SE	
One year before initiation of regular nebulised hypertonic saline	44	2.18	2.00	1.69	0.25	
One year after initiation of regular nebulised hypertonic saline	44	1.20	1.00	1.29	0.19	
p-value						<0.001

**Figure 1:**
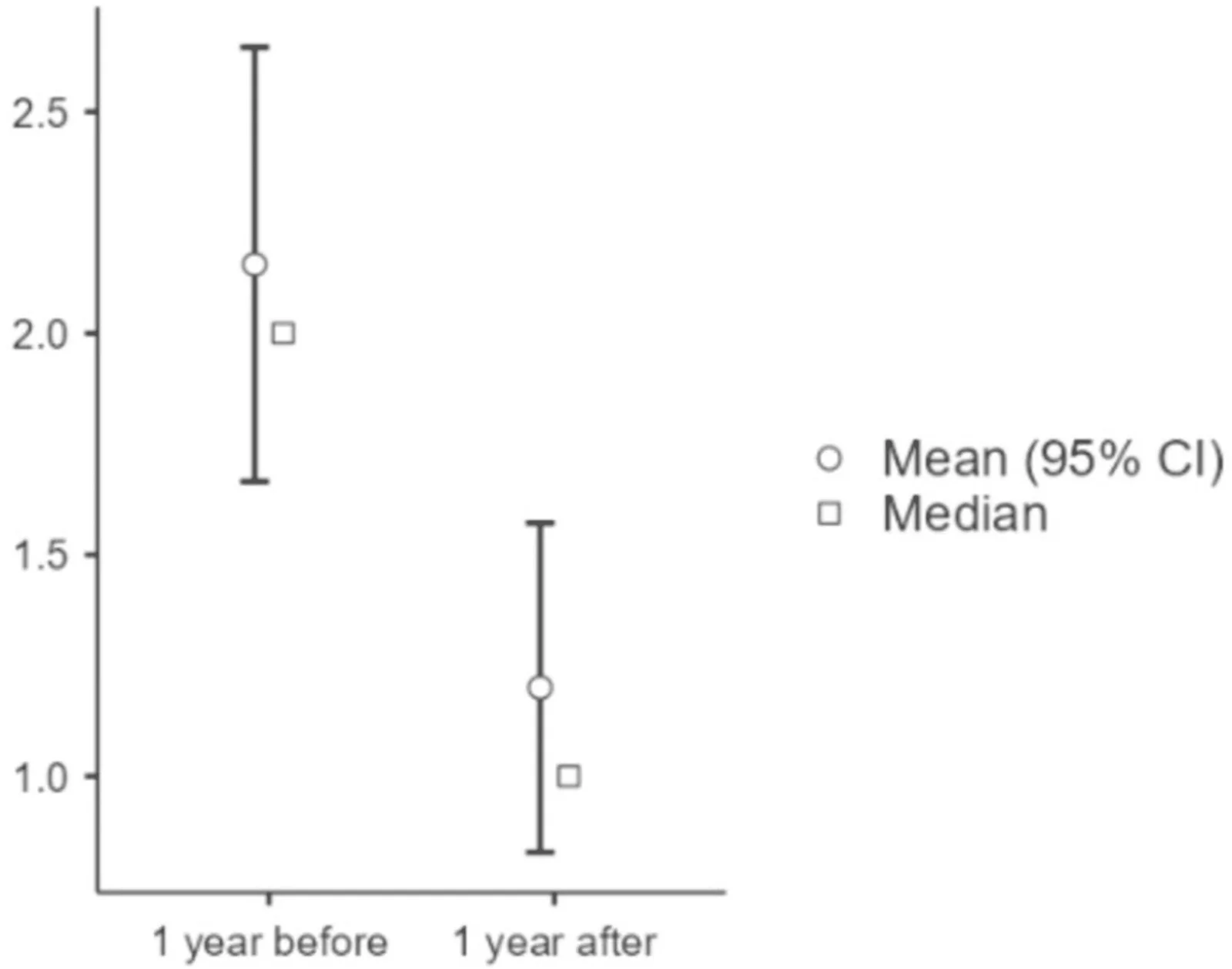
Exacerbation of asthma recorded one year before and after initiation of regular nebulised hypertonic saline

The average number of hospital admissions due to an acute exacerbation of asthma was 0.43 across the 44 patients in the one year before initiation of HS. In the year after, the number of hospital admissions for the same reason was 0.15 (Table 2). Figure 2 demonstrates the reduction in the mean number of recorded hospital admissions one year prior to and one year after starting treatment. The maximum number of admissions in the year before was three and reduced to two in the year after treatment initiation. The combined number of hospitalisation appearances reduced from 19 to 7, a 63% reduction. The mean change in hospitalisation frequency was -0.273 (95% CI: -0.473, -0.0721). 

**Table 2:** Hospital admissions due to exacerbation of asthma recorded one year before and after initiation of regular nebulised hypertonic saline

Number of admissions due to exacerbation of asthma	N	Mean	Median	SD	SE	
One year before initiation of regular nebulised hypertonic saline	44	0.43	0	0.72	0.11	
One year after initiation of regular nebulised hypertonic saline	44	0.15	0	0.42	0.06	
p-value						0.006

**Figure 2:**
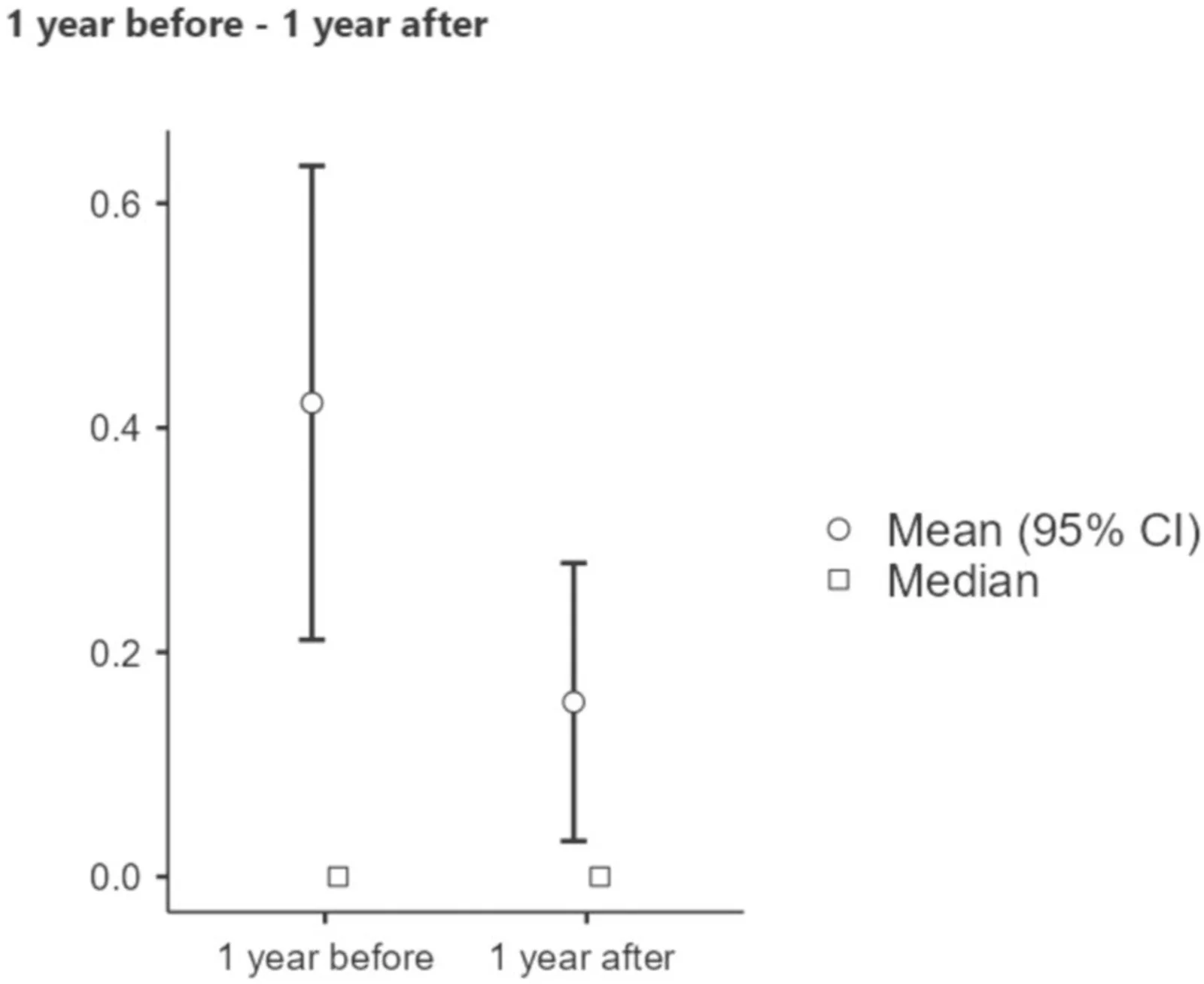
Number of hospital admissions due to exacerbation of asthma recorded one year before and after initiation of regular nebulised hypertonic saline

Following the initiation of nebulised HS treatment, a total of 11 (25%) patients were started on biologic treatment. Of these, six patients were started on benralizumab, four on tezepelumab, and one on a combination of benralizumab and omalizumab.

## Discussion

This retrospective cohort study assesses the benefit of nebulised HS on patients with T2-low and T2-high asthma in the context of reducing the number of hospital admissions and number of exacerbations.

There are currently no dedicated trials assessing the tolerability of nebulised HS in this patient group, with literature primarily comprised of single dose/acute challenge safety studies. This is of importance because the safety profile seen following a single dose may not fully reflect tolerability in long-term use. The literature on dedicated studies around maintenance nebulised HS is in the context of other diseases, such as cystic fibrosis and chronic obstructive pulmonary disease.

Nevertheless, a small study investigating the acute effects of nebulised HS on mucociliary clearance in adults with moderate-to-severe asthma found that participants who inhaled 7% HS experienced a substantial increase in mucociliary clearance, with no recorded deterioration in lung function up to 30 minutes after administration. However, this improvement was not maintained when participants were retested four hours later. This acute increase in mucociliary clearance could be explained by HS increasing airway surface liquid and facilitating mucus clearance, supporting its use as a treatment for clearing mucus secretions. However, the study found no sustained benefit to mucociliary clearance and therefore Bennett et al. concluded the data did not provide evidence to support the use of HS as a maintenance therapy based on there being no evidence for sustained improvements in mucociliary clearance alone [13].

In contrast, our data offer a different perspective as it looked at the long-term use of nebulised HS rather than the physiological response to a single dose. Of the 49 patients who underwent an initial HS trial, 48 (98%) tolerated the trial and were subsequently initiated on treatment. Of these, 44 (91.7%) continued treatment for at least one year. The high level of treatment persistence suggests that nebulised HS was generally acceptable and tolerable as a longer-term treatment in this cohort; however, the reasons for continued treatment were not formally assessed.

The differences between the study populations should also be considered. Bennett et al. studied a small cohort of eight young, predominantly stable female patients with well-controlled moderate-severe asthma [13], whereas the present study included a substantially larger and older cohort, with an age range of 29-89 years. Furthermore, one-quarter of the present cohort subsequently received biologic therapy, reflecting the severity and complexity of asthma within this population.

While the findings of Bennett et al. do not support HS as a maintenance therapy, the effect of HS on airway hydration and mucus clearance may be transient, such that repeated administration could provide benefit [13]. Therefore, the findings of Bennett et al. and the results outlined in our study may be viewed as complementary rather than contradictory: while Bennett et al. provide evidence for an acute physiological effect of HS, the present study provides preliminary observational evidence regarding the tolerability of HS and potential longer-term clinical associations. Our findings suggest that HS may have a potential role as a long-term treatment. However, continued use does not necessarily demonstrate clinical effectiveness, and further research is required to establish whether long-term use of HS results in measurable improvements in lung function, exacerbation rates, symptom control, or quality of life.

De la Fuente et al. assessed sputum induction using HS in 64 patients with varying severities of asthma. The procedure was discontinued in 11.6% of patients because of adverse effects, including a 10-20% fall in FEV1; however, HS was well tolerated by the majority of participants. Sheehan et al. also looked at the safety of airway clearance techniques, sputum induction, and bronchoscopy in severe asthma. The study reports on 117 procedures involving the use of 7% HS, which were generally well tolerated. Having said this, bronchospasms requiring the administration of salbutamol occurred in 5% of the procedures using HS [14].

The high tolerability of using nebulised HS found in the present study is consistent with previous literature suggesting that HS can be administered safely to patients with asthma. However, it is important to recognise that the data provided by De la Fuente et al. and Sheehan et al. primarily assess the short-term safety of using HS during procedures, whereas the data from our study provide information on the long-term tolerability of nebulised HS as a treatment [14,15]. In our study, 98% of patients tolerated the initial HS trial and were subsequently initiated on treatment, with 91.7% of patients continuing treatment for at least one year. This suggests that nebulised HS was well tolerated over the longer term in this cohort.

An important finding of the present study was the 45% reduction in the mean number of recorded asthma exacerbations following initiation of HS treatment, with the mean number of exacerbations per year decreasing from 2.18 to 1.20. There are several possible explanations to consider when interpreting this finding. Firstly, it is possible that HS may have provided a genuine clinical benefit. By increasing airway surface liquid, HS may facilitate the clearance of mucus from the airways, potentially reducing mucus obstruction and contributing to improved airway function and a reduction in asthma exacerbations. However, the present study did not directly assess mucociliary clearance, mucus burden, or airway plugging, and therefore this proposed mechanism cannot be confirmed from the data collected [16].

Secondly, medication changes occurring alongside the initiation of HS may have contributed to the reduction in exacerbations observed. Of the patients established on HS treatment, 25% were subsequently commenced on biologic therapy such as benralizumab, tezepelumab, and omalizumab. As biologic therapies are associated with reductions in asthma exacerbations, their introduction represents an important potential confounding factor and means that the observed reduction cannot be attributed to HS alone.

Our findings are consistent with those of Priel et al., who conducted a retrospective cohort study of patients with severe asthma and severe asthma/CF overlap experiencing recurrent exacerbations and referred to a tertiary asthma centre [17]. Their study assessed exacerbation frequency before and after initiation of regular nebulised HS and demonstrated a statistically significant reduction in exacerbations in the year following treatment initiation (p = 0.0043). Of the 29 patients included in their analysis, only one discontinued treatment due to poor tolerability [17]. The cohort included a mixture of CFTR mutation carriers and non-carriers. The reduction in exacerbation frequency observed by Priel et al. is consistent with the statistically significant reduction observed in the present study. These findings are consistent with a potential clinical benefit of using regular nebulised HS in selected patients with severe asthma. However, both studies were retrospective and used uncontrolled before-and-after comparisons, limiting the ability to determine whether the observed reductions were attributable to HS treatment alone.

It is important to acknowledge that although our study demonstrated a reduction in asthma exacerbations following HS initiation, evidence from other chronic airway diseases does not consistently demonstrate a reduction in exacerbation frequency. In the recently published Chlorthalidone in Chronic Kidney Disease (CLEAR) trial in non-cystic fibrosis bronchiectasis, 52-week treatment with HS did not significantly reduce pulmonary exacerbations compared with standard care [18]. These findings highlight the possibility that the clinical effects of HS may be disease- or phenotype-specific and that improvements in mucus clearance do not necessarily translate into fewer exacerbations.

A further important finding of our study was the reduction in hospital admissions due to asthma exacerbations following initiation of regular nebulised HS. The mean number of hospital admissions decreased from 0.43 per patient in the year prior to HS initiation to 0.15 per patient in the following year, representing an approximate 63% reduction. Interestingly, the reduction in hospital admissions was greater than the reduction in overall exacerbations. This may suggest that fewer exacerbations were severe enough to require hospital admission, although individual patient data would be required to establish this.

There is limited published evidence directly assessing the effect of long-term nebulised HS on hospital admissions in asthma. Much of the existing literature, as previously mentioned, focuses on short-term physiological outcomes, safety and mucociliary clearance rather than longer-term clinical outcomes such as frequency of exacerbations and patients requiring hospital admission. The reduction in admissions observed in our study is therefore of interest, particularly given the potential clinical significance of reducing healthcare utilisation in patients with severe asthma. Evidence from other respiratory conditions provides some biological and clinical rationale for this finding, although it cannot be directly extrapolated to asthma. For example, Zhang et al. investigated the proposed mechanism of HS in bronchiolitis rather than asthma, providing evidence regarding its potential effects on airway hydration and mucus clearance [19]. Similarly, a 2023 study investigating nebulised HS in patients with bronchial hypersecretion reported a reduction in hospitalisations following treatment initiation, although the study population was predominantly composed of patients with bronchiectasis and therefore the findings cannot be considered direct evidence of an effect in asthma [9].

Taken together, these findings provide support for the hypothesis that regular nebulised HS has clinically relevant effects in patients with chronic airway mucus hypersecretion. However, the evidence remains indirect, and the present study cannot determine whether the reduction in hospital admissions was a direct consequence of HS treatment. In addition to biologic therapy and other changes in asthma management, several other factors may have influenced the observed results. These include regression to the mean, as well as changes in healthcare utilisation and the recording of exacerbations and hospital admissions. Further prospective controlled studies are therefore required to determine whether regular nebulised HS independently reduces asthma exacerbations and hospital admissions.

Strengths and limitations

A strength of this study was that data were collected across two hospitals, which increased the sample size. A standardised data collection key was used across all data collection, reducing inter-observer variability in how outcomes were recorded. Another strength is the standardised comparison period, allowing direct comparison between pre- and post-nebulised HS; this reduces the risk of bias from variable follow-up duration. A notable strength is that this study captured exacerbations requiring hospitalisation as well as those which did not, which provides a more realistic picture of this treatment's impact on disease control and exacerbation burden.

We must acknowledge that this study has several limitations. Firstly, the small sample size following application of the exclusion criteria meant that only 44 patients were included, which limits the statistical power of this study. It also reduces the confidence in the generalisability of the findings to the wider population with the same diagnosis. The patients were collated from two predominantly rural hospitals, which improved sample size, but these hospitals were from within the same health board where the local practice is consistent, which may limit generalisability to larger, tertiary centre patient groups. As a retrospective study, there are limitations in terms of the potential influence of confounding factors, which could not be controlled. The data collection relied on electronic health records, which may be subject to inaccuracies or inconsistent documentation, and relied on correct coding of diagnosis/symptoms in the GP records. There were no formally outlined criteria/definition for the bronchial hypersecretion phenotype, which may limit the reproducibility of this study amongst other cohorts. This study did not have a comparison group for patients not receiving nebulised HS, and so the results cannot be solely attributed to the intervention as there may be influence from natural variation in disease course or other concurrent treatment alterations.

Clinical implications

Twice daily nebulised HS was well tolerated in our cohort and was associated with statistically significant reductions in both exacerbation frequency and hospital admissions in patients with T2-low and T2-high asthma with bronchial hypersecretion phenotype. This therefore suggests that regular nebulised HS may be a useful adjunct in the management of this patient group, but findings should be interpreted in the context of the discussed limitations of this study. However, it is important to note the practical challenges, which may be associated with this treatment. Twice daily nebulisation adds considerable treatment burden to these patients, who are most likely already on complex treatment regimens, which may have implications for adherence. As the benefit of nebulised HS relies on continued use, any lapse in adherence may lead to loss of benefit, since the effect is unlikely to be durable after discontinuation. The supervised nebulised HS trial, involving pre- and post-treatment spirometry, requires specialist clinic time and access to trained staff, which could restrict treatment availability.

Future research

Future research should focus on mechanism-based phenotyping of asthma with bronchial hypersecretion and direct comparison between groups. Translational work is needed to define advanced phenotypes and biomarkers, including CT-driven mucus plug scoring and sputum rheology and percentage solids [20,21]. This could identify patients most likely to benefit from HS and provide objective measures of treatment response in both T2-low and T2-high disease. Research could also address the lack of a durable effect through sustained-release osmolyte formulations, which may prolong airway hydration between doses, or through combination therapy with biologics to assess whether HS adds benefit when mucus plugging persists despite targeted treatment [22]. Improving tolerability and safety is also important, and future trials may evaluate hyaluronic acid adjuncts, alongside optimal concentration testing to establish the lowest effective concentration that limits bronchoconstriction. In conclusion, large prospective, controlled studies incorporating these elements would clarify which patients benefit most, and by what mechanism.

## Conclusions

Long-term nebulised HS was well tolerated in this cohort of patients with T2-low and T2-high asthma with a bronchial hypersecretion phenotype. Treatment initiation was associated with significant reductions in asthma exacerbations and hospital admissions over the following 12 months. However, this was a retrospective observational study, so we cannot establish causality, and confounding factors such as the initiation of biologic therapy may have had an influence. Further prospective controlled studies are needed to establish its clinical efficacy and potential role as an adjunctive treatment in this patient population.
